# Residual analysis of chitosan-based agronanofungicides as a sustainable alternative in oil palm disease management

**DOI:** 10.1038/s41598-020-79335-6

**Published:** 2020-12-18

**Authors:** Farhatun Najat Maluin, Mohd Zobir Hussein, Nor Azah Yusof, Sharida Fakurazi, Zainol Maznah, Abu Seman Idris, Nur Hailini Zainol Hilmi, Leona Daniela Jeffery Daim

**Affiliations:** 1 Institute of Advanced Technology, Universiti Putra Malaysia, 43400 UPM Serdang, Selangor Malaysia; 2 Department of Chemistry, Faculty of Science, Universiti Putra Malaysia, 43400 UPM Serdang, Selangor Malaysia; 3 Department of Human Anatomy, Faculty of Medicine and Health Sciences, Universiti Putra Malaysia, 43400 UPM Serdang, Selangor Malaysia; 4 Malaysian Palm Oil Board (MPOB), 6, Persiaran Institusi, Bandar Baru Bangi, 43000 Kajang, Selangor Malaysia; 5 Sime Darby Technology Centre Sdn. Bhd., UPM-MTDC Technology Centre III, Lebuh Silikon, Universiti Putra Malaysia, 1st Floor, Block B, 43400 Serdang, Selangor Malaysia

**Keywords:** Nanoscale materials, Analytical chemistry, Environmental chemistry, Plant sciences, Environmental sciences

## Abstract

The nanoformulations of pesticides have shown great interest from many parties due to their slow release capability and site-specific delivery. Hence, in this work, a new nanoformulation of a fungicide, namely chitosan-hexaconazole nanoparticles with a mean diameter size of 18 nm was subjected to the residual analysis on oil palm tissue, leaf and palm oil (crude palm oil and crude palm kernel oil) using a quick, easy, cheap, effective, rugged and safe (QuEChERS) method coupled with the gas chromatography–micro electron capture detector (GC–µECD). The chitosan-hexaconazole nanoparticles were applied using the trunk injection method at 4.5 g a.i./palm (standard single dose) and 9.0 g a.i./palm (double dose). The fungicide residue was analyzed at 0 (6 h after application), 1, 3, 7, 14, 30, 60, 90, and 120 days after treatment. The palm oil matrices; the crude palm oil (CPO) and crude palm kernel oil (CPKO) were found to be residue-free. However, it was observed that high accumulation of the fungicide in the stem tissue and leaf after the treatment using the chitosan-hexaconazole nanoparticles, which is good for better bioavailability for the treatment of the fungi, *Ganoderma boninense*. The dissipation kinetic at double dose treatment in the tissue and leaf was found to govern by the second-order kinetic with half-lives (t_1/2_) of 383 and 515 days, respectively.

## Introduction

Oil palm (*Elaeis guineensis*) is originates from the West African tropical rainforest. The average productive span-life of an oil pam is around 25–30 years in which each tree will carry 8–12 bunches of fruit each year^1^. The palm is able to grow to more than 30 feet and start producing fruit bunches from the age of 3 years after planting. Palm oil is emerging as the most productive vegetable oilseed crop due to its high oil yield^2^. Palm oil’s yield can be extracted from the fibrous mesocarp of the fresh fruit bunches (called as crude palm oil, CPO) or from the palm kernel, the seed of the endocarp shell (called as crude palm kernel oil, CPKO). Compared to the other world’s leading oilseeds crops including soybean, sunflower, and rapeseed, one hectare of an oil palm plantation can produce up to tenfold oil^2^. Oil palm, however, has been threatened by the lethal disease of basal stem rot disease caused by a pathogenic *Ganoderma boninense* fungus and has consequently become a major concern in the oil palm industry^3–5^.

68 million tonnes of palm oil, 54 million tonnes of soybean oil, 25 million tonnes of rapeseed oil, and 19 million tonnes of sunflower oil are the world’s major oilseed production in 2017^6^. In addition, palm oil contributed to one-third of the world’s oil and fats production by 34%^7^. Hexaconazole was used to combat basal stem rot disease in the oil palm plantation^8,9^. The use of conventional hexaconazole, however, has significant adverse environmental effects and as it is dissipated and leached, it is a threat to both terrestrial and aquatic life^10,11^. The high fungicide residue has also been shown to increase soil acidity^12^. Therefore, urge for an alternative, sustainable approach to ensure that instead of leaching out and drainage to the surrounding soil and river, the applied fungicide was distributed to targeted pathogenic fungus.

In this study, a new nanoformulations, chitosan-based agronanofungicides consisting of chitosan (nanocarrier) and hexaconazole (active ingredient, a.i.) is used in the oil palm management as it is proved to have high antifungal activity on *Ganoderma boninense*, a pathogenic fungal that leads to basal stem rot disease^13^. The use of chitosan as a nanocarrier has been widely researched, in which the nanocarrier system enabled the attachment, encapsulation, and entrapment of agricultural active ingredients to develop an effective formulation of the nanodelivery system^14^. The nanodelivery system offers controlled release properties with high efficacy and potency as the fungicides can reach the target fungus more effectively, compared to their counterparts^15^. The formulations also aim to improve the solubility and stability, minimizing volatilization, enhance uptake as well as reduce their toxicity level, thus minimizing their negative impacts on the environment^16^. In addition, chitosan offers non-toxicity, biocompatibility and known for its ability to control or reduce the spreading of disease in the plant by inhibiting pathogens and enhance the plant defense mechanism^17^.

Research on the determination of pesticide residues in food and plant matrices has increased significantly as the use of pesticides in crop management is inevitable. Concerning that, a quick, easy, cheap, effective, rugged and safe (QuEChERS) method has become a forerunner in this field, offering rapid analysis, inexpensive, and broad applicability^18–20^. In this method, the pesticide is extracted by centrifugation of plant or food-based material with acetonitrile solvent. Magnesium sulfate (MgSO_4_) and sodium chloride (NaCl) are then added to eliminate the water in acetonitrile. The extracted liquid is then subjected to the dispersive solid-phase separation (d-SPE) with a primary-secondary amine (PSA), graphitized carbon black (GCB), octadecyl (C_18_) and MgSO_4_. The role of PSA is to remove polar interfering substances, including organic acids, pigments, sugars, and fatty acids. GCB and C_18_ are for the removal of sterols (i.e., chlorophyll) and non-polar interfering substances (i.e., lipids), respectively^21^.

Herein, we define our work on the assessment of the fungicide residues, namely hexaconazole, due to the application of our newly developed agronanofungicide, chitosan-hexaconazole nanoparticles (18 nm, mean particle size diameter determined via HRTEM) for the treatment of BSR disease caused by *G. boninense*. We have chosen four samples, namely oil palm matrices, including leaf, tissue, crude palm oil, and crude palm kernel oil. The samples were then subjected to the QuEChERS extraction method and been analyzed using the gas chromatography-micro electron capture detector (GC-µECD). Under the international pesticide legislation, the detection of pesticide residues in food is a key step in the control and authorization of pesticides^22^. Hence, the elimination of hexaconazole residues in palm oil is a crucial step in preventing any food contamination that could have a negative impact on public health. The nanoformulations also helps to improve the uptake, bioavailability, and internalization stability of fungicide within the plant. Hence, the accumulations, dissipation kinetic, and half-lives (t_1/2_) of chitosan-hexaconazole nanoparticles in the stem tissue and leaf were evaluated. The proposed method was validated using matrix effect, selectivity, sensitivity (limits of detection and quantification), and efficiency of the extraction (recovery studies).

## Results and discussion

### Matrix effect, the limit of detection and quantification

**Figure 1:**
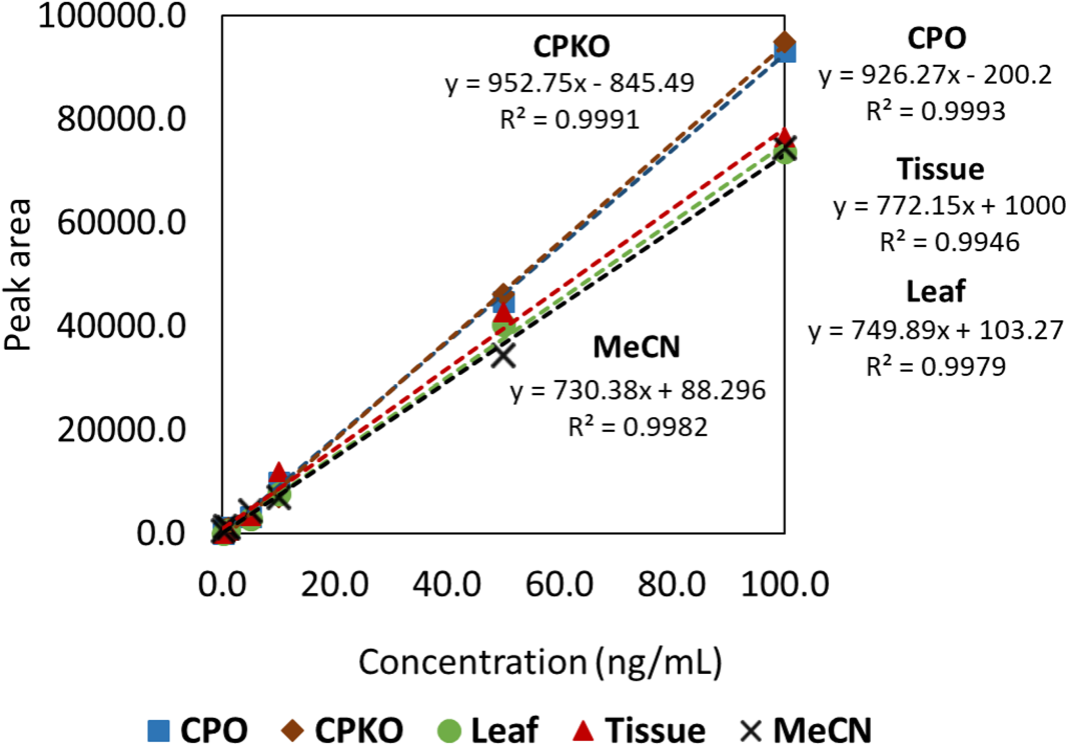
Solvent (MeCN) calibration curve and matrix-matched calibration curve in CPO, CPKO, leaf, and tissue.

**Figure 2:**
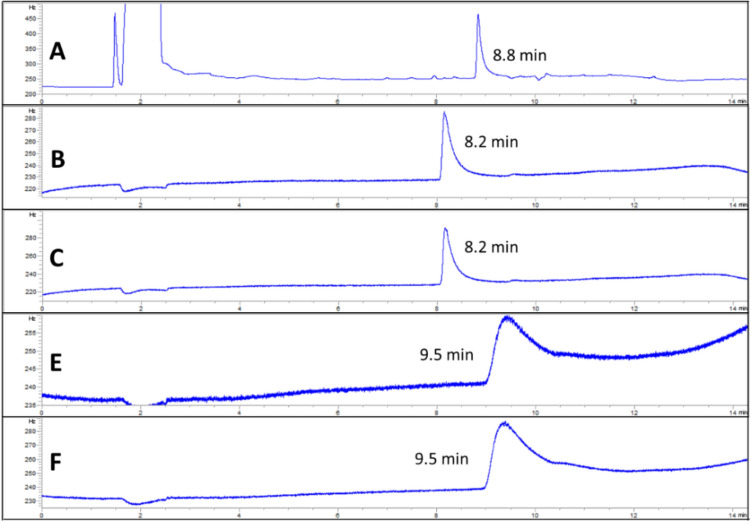
Hexaconazole (5.0 ng/mL) peak comparison in the solvent (**A**) MeCN and matrix of (**B**) CPO, (**C**) CPKO, (**D**) leaf and (**E**) tissue.

As shown in Fig. 1, all calibration curves in the solvent and matrix-matched showed excellent linearity with R^2^ > 0.99. IUPAC defined sensitivity as equivalent to the calibration curve^23^. Hence, indicating higher sensitivity in the matrix of CPO and CPKO as the higher slope was observed in CPO and CPKO matrix calibration curve. Moreover, lower LODs and LOQs were found in CPO-matrix with 1.6 and 5.0 ng/mL, respectively, and CPKO-matrix with 1.9 and 5.8 ng/mL, respectively (Table 1). The value for leaf-matrix with LOD and LOQ of 2.9 and 8.7 ng/mL, respectively, and tissue-matrix with LOD and LOQ at 4.6 and 14.0 ng/mL, respectively were obtained. In addition, the peak of hexaconazole in MeCN was observed at 8.8 min. A slight shift in the peak of the hexaconazole peak was observed in the CPO-matrix and CPKO-matrix at 8.2 min. On the other hand, the peak for leaf-matrix and tissue-matrix shifted the retention time to 9.5 min (Fig. 2). This indicated the matrix effect in the peak of hexaconazole, where the matrix effect is the difference between the response of the same analyte at the same concentration in the standard solvent solution and the plant/food-based matrix solution**.**

**Table 1:** Limit of detection (LOD), limit of quantification (LOQ), retention time (RT) and percentage of matrix effect (ME %) of CPO, CPKO, leaf, and tissue of oil palm.

Matrix-matched calibrations	LOD (ng/mL)	LOQ (ng/mL)	RT (min)	ME (%)
CPO	1.6	5.0	8.2	21.1
CPKO	1.9	5.8	8.2	23.3
Leaf	2.9	8.7	9.5	2.6
Tissue	4.6	14.0	9.5	5.9

The matrix effect (ME) percentage was then further quantified and listed in Table 1. The positive value %ME or higher slope of matrix-matched calibration indicates analyte signal enhancement, whereas the negative value %ME or lower slope of matrix-matched calibration represents analyte signal suppression induced by the matrix^24,25^. The value of %ME greater than 20% was reported as having a significant effect on the quantitative analytical measurement, while the value below 20% can be considered having no matrix effect^26^. Hence, minor matrix-enhancement effects were observed in CPO and CPKO, with %ME at 21.1% and 23.3%, respectively. No matrix effect was observed in tissue and leaf. The effect might be due to the presence of the lipids and fatty acid in palm oil matrices, which caused interference in the measurement of the analyte^27^. Therefore, highlighting the importance of constructing a matrix-matched calibration curve for the determination of hexaconazole in an unknown sample. The selectivity of the system in all matrix solutions showed high selectivity as no other noticeable interference peak signal was observed at the scanning time up to 14.5 min (Fig. 2). Moreover, the excellent linearity of the curve in all the matrix solutions indicates that it is sensitive enough for the quantification of hexaconazole in the real sample monitoring.

### Evaluation of the QuEChERS method

The method was validated by a recovery study by spiking with 1.0, 5.0, 10.0, and 50.0 ng/g of hexaconazole and comparing the analyte peak area of the spiked sample with the standard matrix-matched calibration solution. The recoveries obtained in all the four matrix solutions are above 100%, and the RSD values were below 3.0% (Table 2). The acceptable range recovery percentage recorded is between 70%–120%, and the reproducibility of the RSD value is below 20%^28^. Hence, both recoveries and RSD met the performance requirements of the method in all the matrix solutions, indicating the precision and consistency of the proposed QuEChERS method.

**Table 2:** Recoveries of hexaconazole from matrix solution of CPO, CPKO, leaf, and tissue of oil palm.

Spike concentration (ng/g)	CPO		CPKO		Leaf		Tissue	
	recovery (%)	RSD (%)	recovery (%)	RSD (%)	recovery (%)	RSD (%)	Recovery (%)	RSD (%)
5.0	102.5	2.6	105.3	1.6	103.2	0.8	108.1	3.2
10.0	103.2	3.3	104.3	3.3	112.8	1.2	102.4	1.9
50.0	109.6	1.2	107.0	4.2	109.3	5.0	115.8	5.1
100.0	109.3	2.5	106.3	5.3	117.6	1.2	113.5	0.5

### Residual analysis of palm oil matrices

All the extracted 90 samples of CPOs and CPKOs were subjected to the residual analysis of hexaconazole. No peak of hexaconazole was observed in both single-dose and double-dose of hexaconazole at 0, 1, 3, 7, 14, 30, 60, 90, and 120 days. This indicated zero hexaconazole residue on the palm oil matrices of CPO and CPKO (Table 3). The study included the development of a new fungicide to ensure that there is no residue of a toxic fungicide on palm oil in such a way that it is safe for consumers. It is worthy to note that, the acceptable daily intake levels of hexaconazole according to the joint Food and Agriculture Organisation (FAO) and World Health Organisation (WHO) are established at 0.005 mg/kg of body weight/day^29^.

**Table 3:** The accumulation of chitosan-hexaconazole nanoparticles and conventional hexaconazole residues found on the fruits of oil palms.

Days after treatment	The concentration of chitosan-hexaconazole nanoparticles residue (ng/g)				Reference
	CPO sample		CPKO sample		
	Single dose (4.5 g a.i./palm)	Double dose (9.0 g a.i/palm.)	Single dose (4.5 g a.i./palm)	Double dose (9.0 g a.i./palm)	
0 (6 h)	ND	ND	ND	ND	Current work
1	ND	ND	ND	ND	
3	ND	ND	ND	ND	
7	ND	ND	ND	ND	
14	ND	ND	ND	ND	
30	ND	ND	ND	ND	
60	ND	ND	ND	ND	
90	ND	ND	ND	ND	
120	ND	ND	ND	ND	
**The concentration of conventional hexaconazole nanoparticles residue (ng/g)**					
0 (6 h)	ND	ND	ND	ND	31
1	ND	ND	ND	ND	
3	ND	ND	ND	ND	
7	ND	ND	ND	ND	
14	ND	ND	ND	ND	
21	ND	ND	ND	ND	
30	ND	ND	ND	ND	
70	ND	ND	ND	ND	

*ND* not detected.

### Residual analysis of oil palm tissue and leaf

**Figure 3:**
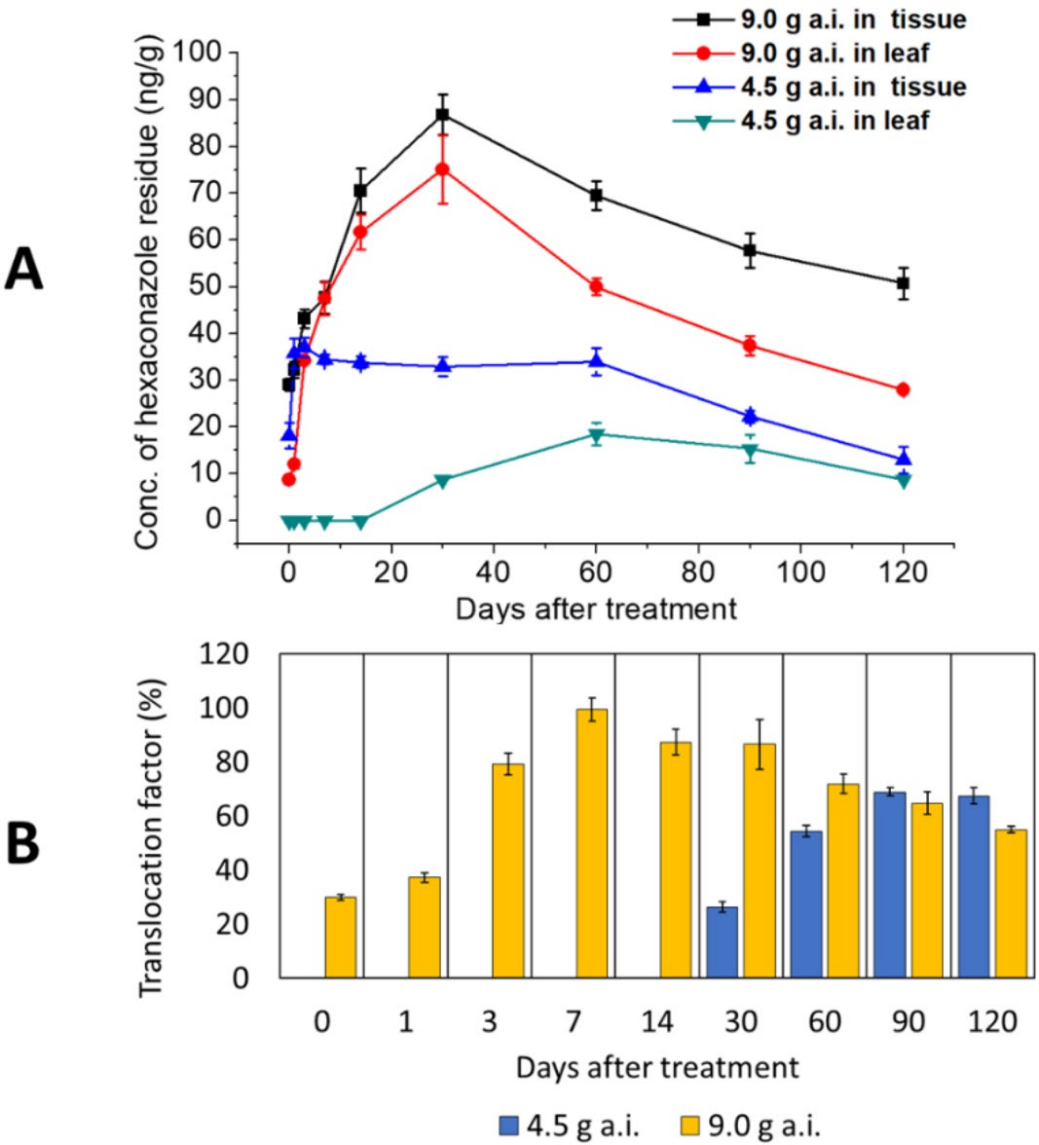
(**A**) Hexaconazole accumulation found in leaf and tissue and (**B**) their percentage translocation factor from tissue to leaf, where the error bars represent the standard deviation of the mean.

Residual hexaconazole collected from the oil palm leaf is shown in Table 4 and Fig. 3A. At the single dose, the residue in leaf was only detectable on day-30 onwards, where the highest concentration of hexaconazole was observed on day-60. While for the double dose, the residue in leaf was already detectable as early as 6-h after treatment. The concentration of residual hexaconazole in the leaf was found to increase gradually until day-30 and started to decrease slowly on day-60 onwards.

**Table 4:** The accumulation of chitosan-hexaconazole nanoparticles and conventional hexaconazole residue found on the leaf, where different letters in the same column indicate significant differences between means (p ≤ 0.05) according to Tukey’s test.

Leaf sample					
Days after treatment	The concentration of chitosan-hexaconazole nanoparticles residue (ng/g)		Days after treatment	The concentration of conventional hexaconazole residue (ng/g)^31^	
	Single dose (4.5 g a.i./palm)	Double dose (9.0 g a.i/palm.)		Single dose (4.5 g a.i./palm)	Double dose (9.0 g a.i./palm)
0 (6 h)	ND	< 8.7^a^	0 (6 h)	128.0 ± 4.0^a^	216.0 ± 5.0^a^
1	ND	12.0 ± 0.9^b^	1	97.0 ± 12.0^b^	171.0 ± 7.0^a^
3	ND	34.2 ± 0.4^c^	3	52.0 ± 1.0^b^	91.0 ± 4.0^b^
7	ND	47.4 ± 3.5^d^	7	59.0 ± 3.0^b^	65.0 ± 3.0^b^
14	ND	61.7 ± 3.7^e^	14	44.0 ± 1.0^b^	56.0 ± 6.0^c^
30	< 8.7^a^	75.1 ± 7.3^e^	21	28.0 ± 1.0^b^	62.0 ± 2.0^d^
60	18.5 ± 2.4^b^	50.0 ± 1.8^d^	30	ND	29.0 ± 3.1^c^
90	15.3 ± 3.0^b^	37.4 ± 2.1^c^	70	ND	ND
120	< 8.7^a^	27.9 ± 0.6^c^			

*ND* not detected.

In the tissue sample (Table 5 and Fig. 3A), a sharp increase in the accumulation of hexaconazole was found at a single dose on the 1st day compared to 6-h after treatment. The plateau in the accumulated hexaconazole was then observed until the 60th day. Moreover, the highest concentration of the accumulated hexaconazole in tissue was found on day-30 at the double-dose treatment. As shown in Fig. 3A, the same trend was observed in the uptake of hexaconazole on leaf and tissue at the double-dose treatment. Notably, it took 30 days for the single-dose to be translocated to the upper part of 10–13 feet of oil palm.

**Table 5:** The accumulation of chitosan-hexaconazole nanoparticles residue found on tissue, where different letters in the same column indicate significant differences between means (p ≤ 0.05) according to Tukey’s test.

Days after treatment	Tissue sample	
	The concentration of chitosan-hexaconazole nanoparticles residue (ng/g)	
	Single dose (4.5 g a.i./palm)	Double dose (9.0 g a.i./palm)
0 (6 h)	18.1 ± 2.8^a^	29.0 ± 1.3^a^
1	35.7 ± 3.2^b^	32.2 ± 1.7^a^
3	37.0 ± 2.0^b^	43.2 ± 2.0^b^
7	34.5 ± 1.0^b^	47.7 ± 3.4^b^
14	33.8 ± 1.3^b^	70.6 ± 4.8^c^
30	32.9 ± 2.0^b^	86.8 ± 4.3^d^
60	34.0 ± 2.9^b^	69.5 ± 3.1^c^
90	22.2 ± 1.3^c^	57.7 ± 3.7^c^
120	12.9 ± 2.8^d^	50.7 ± 3.4^b^

*ND* not detected.

The translocation factor (TF) of hexaconazole from the stem tissue to the leaf was then quantitated using Eq. (1)^30^.

1 $$TF \left( \% \right) = (residual\,found\,on\,leaf/residual\,found\,on\,tissue) \times 100 .$$

At a single-dose treatment, > 50% of the chitosan-hexaconazole nanoparticles were translocated from stem to the leaf on day-60 and onwards. Moreover, at the double dose treatment, the gradual increase of TF was found from day-0 until day-7 with 100% of the chitosan-hexaconazole nanoparticles was translocated from stem to the leaf at day-7 (Fig. 3B). Even on the final day of the study, at 120-day after treatment, a good amount of hexaconazole releases from the chitosan-hexaconazole nanoparticles were still can be traced to be translocated from stem to the leaf (> 50%).

The results indicated the ability of chitosan-hexaconazole nanoparticles to survive and circulate in the crop longer than the conventional pure hexaconazole equivalent. Previous work has reported that oil palm treated with the conventional hexaconazole at 9.0 g AI/palm can only be detected up to the day-30^31^. We believe that the slow-release properties of the chitosan-hexaconazole nanoparticles might contribute to this. As reported in our earlier work, the release time of chitosan-hexaconazole nanoparticles is six-time longer compared to its counterpart^13^. In vitro release of hexaconazole at the pH 5.5 (pH of soil) was recorded a prolonged release up to 86 h.

The small size of chitosan-hexaconazole nanoparticles with the mean size of 18 nm was also the crucial factor in the bioavailability enhancement and the efficiency of plant translocation. The pore diameter of the cell wall of plant measured using various techniques has been reported to be generally in the range of 5–20 nm^32,33^. Hence, the nanoparticles of chitosan-hexaconazole can penetrate and permeate into the cell wall through the pore to enter the plasma membrane easily. In addition, the high accumulation and bioavailability of the stem tissue suggest that the nanoparticles of chitosan-hexaconazole are adsorbed into plant tissue via the xylem, in which the mechanism of internalization occurred via systemic xylem mobility^34^. Higher uptake of agronanoparticle in the plant has been widely researched owing to its small size that enables penetration through the cuticle and plant cell wall^35,36^. Moreover, it is also reported the possibility of the cell wall pore enlargement upon interaction with the agronanoparticles, which in turn increases their uptake^37,38^.

### Dissipation kinetics and half-lives

After 30 days of treatment, the residual of hexaconazole seems to dissipated slowly and gradually (Fig. 3A). Hence, to quantitatively study the dissipation residual of hexaconazole, the residual hexaconazole in leaf (9.0 g AI/palm) and tissue (4.5 and 9.0 g AI/palm) were fitted to three different kinetic models and the linear fits of residual concentration curves and their corresponding estimate of the dissipation half-lives (t_1/2_) are presented in Table 6. No data for a single dose at 4.5 g AI /palm leaf can be offered due to insufficient of the residual data. The linear forms in zeroth-order, first-order, and second-order kinetics are presented in Eqs. (2), (3), and (4), respectively^39^, where C_t_ is the residual hexaconazole concentration (ng/g) at time t (day), C_0_ is the hexaconazole concentration at a time zero, and k_d_ is the hexaconazole dissipation rate constant (day^−1^).

2 $$C_{{t{ }}} = { }C_{0} { } - k_{d} \times t,$$

3 $$C_{t} { } = { }C_{0} { } \times \exp ( - k_{d} { } \times t),$$

4 $$C_{t} { } = { }\frac{{C_{0} }}{{1{ } + { }C_{0} { } \times { }k_{d} { } \times t}}.$$

**Table 6:** Dissipation kinetics and half-lives (t_1/2_) of hexaconazole found on the oil palms treated with the agronanofungicides at different a.i. concentrations.

Sample	Dose concentration g of a.i./palm	Zeroth-order		First-order		
		R^2^	Regression line	R^2^	Regression line	t_1/2_ (day)
Tissue	4.5	0.8089	y = − 0.2393x + 43.45	0.8550	y = − 0.0048x + 3.978	147
	9.0	0.9645	y = − 0.4003x + 96.20	0.9867	y = − 0.0026x + 4.621	267
Leaf	9.0	0.9480	y = − 0.5140x + 86.15	0.9926	y = − 0.0047x + 4.611	147

Sample	Dose concentration g of a.i./palm	Second-order				
		R^2^	Regression line		t_1/2_ (day)	
Tissue	4.5	0.8150	y = 0.00050x + 0.0063	110		
	9.0	0.9980	y = 0.00009x + 0.0089	383		
Leaf	9.0	0.9938	y = 0.00020x + 0.0054	515		

The results revealed that the dissipation of hexaconazole of the chitosan-hexaconazole nanoparticles in tissues at the single-dose followed the first-order kinetic with t_1/2_ of 147 days. On the other hand, at the double-dose in tissue and leaf, the dissipation of both followed the second-order kinetic with t_1/2_ of 383 and 515 days, respectively. No t_1/2_ of pure chitosan can be provided due to the laborious and time-consuming procedure. However, we believe that the t_1/2_ of pure chitosan will be much shorter than chitosan-hexaconazole nanoparticles. This is due to the controlled release properties in the nanoparticles.

The findings of this study suggested that nanoparticles of chitosan-hexaconazole were mobilized on the internal part of the oil palm body of stem and leaf only, instead of being translocated to the fruit. This might due to the high levels of un-methylesterified pectin in oil palm fruit, which prevent the chitosan-hexaconazole nanoparticles to enter the fruit. In addition, our previous work on chitosan-hexaconazole nanoparticles revealed its high antifungal activity on *Ganoderma boninense*, a pathogenic fungal that leads to basal stem rot disease in oil palms^13^. Hence, the chitosan-hexaconazole nanoparticles offer a great deal of potential in the management of BSR disease as they effectively control the disease over a long period with residue-free palm oil matrices. This is the ideal desired properties for agronanofungicides for better management of basal stem rot disease of oil palm developed in this work.

## Conclusion

Oil palms were treated with our newly developed agronanofungicide, where the trunk was injected with the new nanoformulations using chitosan-hexaconazole agronanofungicide. It was found that the crude palm oil and crude palm kernel oil is residue-free. Moreover, the high accumulation of fungicide in stem tissue and leaf following treatment with the chitosan-hexaconazole nanoparticles is ideal for improved bioavailability in the treatment of the fungi, *G. boninense*. Double dose kinetic dissipation in tissue and leaf was found to be prevalent in the second-order kinetic with half-lives (t_1/2_) of 383 and 515 days, respectively. The proposed residual analysis method offers rapid and efficient analysis with high sensitivity and selectivity with a meager limit of detection, < 2.0, 3.0, and 5.0 ng/mL for palm oil matrices, oil palm leaf, and oil palm tissue, respectively.

## Materials and methods

### Chemicals and equipment

QuEChERS extraction tube packed with 150 mg MgSO_4_, 50 mg PSA, 50 mg GCB and 50 mg C_18_ was purchased from United Chemical (Bristol, Pennsylvania). Hydrochloric acid (37%) and n-hexane were purchased from Merck (Kenilworth, NJ, USA). Acetonitrile (MeCN) was purchased from System (Selangor, Malaysia). Magnesium sulfate (MgSO_4_) and sodium chloride (NaCl) were purchased from Sigma-Aldrich (St. Louis, MO, USA).

Hexaconazole (95% purity) was used as a standard and was purchased from Changzhou Aiteng (Jiangsu, China). The fungicide of chitosan-hexaconazole nanoparticle with the mean diameter size of 18 nm (measured using high-resolution transmission electron microscopy (HRTEM)) was formulated as previously described and it was in the powder form of the yellowish-white color as previously published^13^. The nanoparticles were formed by loading of the hexaconazole into the chitosan nanocapsule using an ionic gelation method. Sodium tripolyphosphate and tween-80 has been added as its crosslinking agent and stabilizing agent. The fungicide is composed of 85% w/w of chitosan and 15% w/w of hexaconazole.

The sample extracts were analyzed using an Agilent Technologies 7890A Series (Santa Clara, CA, USA) gas chromatograph (GC) equipped with a micro electron capture detector (µECD). The injector mode was splitless, with 2.0 μL injection volume and operated at 250 °C. An Agilent 19091S-433 HP-5MS column (Santa Clara, CA, USA) coated with 5% diphenyl with 30 m length, 250 μm diameter, and 0.25 μm film thickness was used to separate the analytes. Nitrogen gas was used in both carrier gas (1.0 mL/min) and makeup gas (60.0 mL/min). The initial temperature was set at 150 °C for 1 min. Then, the oven was heated to 250 °C at 10 °C/min for 5 min and then to 280 °C at 10 °C/min for 5 min.

### Experimental design

**Figure 4:**
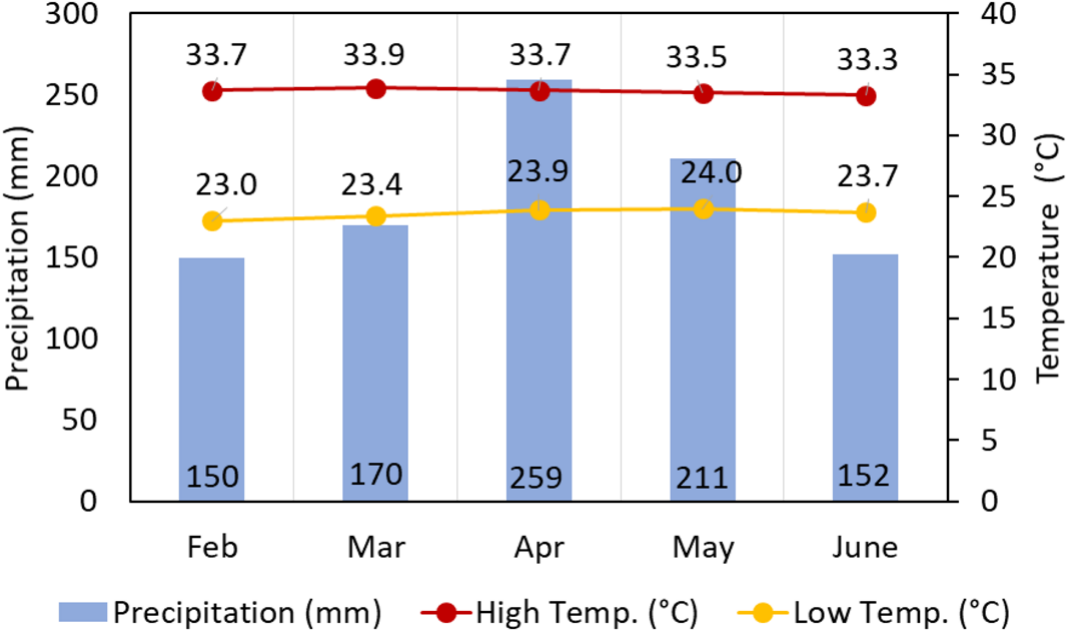
Weather conditions recorded throughout the study.

The field trial was conducted in an oil palm plantation located at Teluk Intan, Perak, Malaysia owned by the Malaysian Palm Oil Board (MPOB) from 26th February 2019 until 26th June 2019. The recorded average temperature and rainfall precipitation in each month is shown in Fig. 4. The trial area, Plot 2A1 with a total of 10.43 hectares was covered with 10–13 feet tall of commercial *Tenera* (*dura* × *pisifera*) species of 13 years old oil palm on peat soil. The field trial experiments were carried out in a randomized complete block design (RCBD) and divided into 9 subplots.

The experiment was conducted in three treatment conditions; (1) control, untreated palm, (2) standard single dose of 4.5 g a.i./palm and (3) double dose of 9.0 g a.i./palm. Each treatment was replicated three times with 10 palms per treatment. A total of 90 oil palm was used throughout this trial. The standard dose was based on the standard procedure on the usage of the conventional hexaconazole for the basal stem rot disease treatment^8^. The application of the agronanofungicide chitosan-hexaconazole nanoparticles on the oil palms was conducted using the Malaysian Palm Oil Board (MPOB) standard procedure, by drilling two holes (20 cm depth) at the trunk of the palm with a motorized driller (diameter 11 mm, length 45 cm). Then, 6 L of the fungicide was injected to the drilled hole (3 L in each hole) using the motorized knapsack sprayer, and lastly, 1 L of the fungicide was sprayed on the trunk and bole of the palm^40^. Prior to the treatments, the chitosan-hexaconazole nanoparticles powder was dissolved in 1.0% v/v of hydrochloric acid solution. The size of the nanoparticles in a solvated state measured using dynamic light scattering (DLS) is below 20 nm as previously published^13^.

The sampling of the fruit bunches, leaf and tissue was done at -1 (before treatment), 0 (6 h after applied), 1, 3, 7, 14, 30, 60, 90 and 120 days after the treatment. The fruit bunches were ensured to be ripe enough for the sampling. Crude palm oil (CPO) was then extracted from the fleshy mesocarp of the fruit and crude palm kernel oil (CPKO) was extracted from the kernel inside the fruit seed. Frond number 17 (calculated from the top) was chosen for the sampling of leaf^31^. Tissue sampling was collected by drilling the palm trunk at 3 feet from the ground with 20 cm depth using the motorized driller (diameter 11 mm, length 45 cm).

### Sample processing

The collected fruit bunches were chopped and the loose fruit was sterilized using a Hirayama HVE-50 Autoclave Sterilizer (Saitama, Japan) for 40 min at 121 °C to avoid contamination. The flesh fibrous mesocarp was then manually separated from the sterilized fruit and heat again at 40 °C for 5 min in the oven before placed on the mini hydraulic hand press machine to extract the CPO^41^. After that, the seed of the fruit was cracked using a hammer in which revealing the kernel inside it. The broken kernel was then further grounded in a blender to obtain a homogenous mixture. Then, 25 g of the ground kernel was weighted into an extraction thimble and the CPKO was extracted using the soxhlet extraction technique using 150 mL n-hexane for 8 h. The extract was then subjected to the rotatory evaporator (Buchi R-205 Rotavapor, Flawil, Switzerland) to eliminate the solvent. Prior to the residue analysis, CPOs and CPKOs were kept in a freezer at 0 °C^41^.

The collected leaf was cleaned and dried in an oven for 4 h at 40 °C. Then, it was cut into smaller pieces, ground using a blender and kept in a freezer at − 20 °C prior to the analysis^31^. The collected tissue was directly kept in a freezer at − 20 °C prior to the analysis.

### Chitosan-hexaconazole nanoparticles extraction method

The extraction of chitosan-hexaconazole nanoparticles in the sample was carried using the QuEChERS analytical procedure^21^ with some modifications. Initially, 5 g of the sample was weighed into 50 mL of a polypropylene centrifuge tube. Then, acetonitrile containing 1% (v/v) of HCl was added (15 mL for CPO and CPKO, 30 mL for leaf and tissue). The mixture was then vortexed for 30 s. A mixture of 4 g of MgSO_4_ and 1 g NaCl was added to promote the partitioning step. The tube then was vortexed again for 30 s and centrifuged for 20 min at 5000 rpm. For the CPO and CPKO samples, the upper layer of acetonitrile was then transferred to another 50 mL of the polypropylene centrifuge tube and freeze out (− 20 °C) for a minimum of 2 h, to precipitate the oil fat (this step was skipped for the leaf and tissue sample). A minimum of 2 h of low-temperature precipitation was found to be the optimum time for fat removal^42^. Then, an aliquot of 1 mL supernatant was transferred into a microcentrifuge tube containing 150 mg MgSO_4_, 50 mg PSA, 50 mg graphitized carbon, 50 mg C_18_ for the dispersive solid-phase extraction (d-SPE) cleanup. The tube extract was then centrifuged for 5 min at 2000 rpm and filtered using polytetrafluoroethylene (PTFE) syringe filter (pore size: 0.22 μm, diameter: 13 mm, hydrophilic membrane). The filtered sample was then subjected to the GC-µECD analysis.

### The matrix-matched calibration curve, the limit of detection (LOD) and the limit of quantification (LOQ)

A stock solution of hexaconazole with a concentration of 10.0 μg/mL was prepared in acetonitrile and serially diluted to working standard solutions of 0.1, 0.5, 1.0, 5.0, 10.0, 50.0 and 100.0 ng/mL. The stock solution was also used for the matrix-matched calibration solution by serial dilution at the same concentrations in the extracted blank CPO, CPKO, tissue and leaf solutions. All solutions were kept in a freezer at -20 °C prior to analysis. To assess the matrix effect (ME) in the calibration, the ME % was then calculated using Eq. (5) ^43^.

5 $$ME\% = \left( {1 - \frac{Solvent\,slope}{{Matrix - matched\,slope}}} \right) \times 100.$$

The limit of detection (LOD) and limit of quantification (LOQ) were calculated using Eqs. (6) and (7), respectively^23^.

6 $$LOD = 3.3 \times \frac{Standard\,deviation\,of\,the\,regression\,line}{{Slope}},$$

7 $$LOQ = 10 \times \frac{Standard\,deviation\,of\,the\,regression\,line}{{Slope}}.$$

### Recovery studies

The accuracy and precision of the method were evaluated using recovery and relative standard deviation (RSD) in 6 replicate measurements. The blank sample of all the matrices (CPO, CPKO, leaf, and tissue) were spiked with hexaconazole at four different concentrations (1.0, 5.0, 10.0 and 50.0 ng/g). The spiked sample was then extracted and analyzed using the method as described above.

### Statistical analysis

Data are presented as mean ± standard deviation and the statistical difference of the parameters was analyzed using the ANOVA and Tukey’s test (*p* ≤ 0.05) using the SPSS software.
